# The Effects of Habitat Type and Volcanic Eruptions on the Breeding Demography of Icelandic Whimbrels *Numenius phaeopus*

**DOI:** 10.1371/journal.pone.0131395

**Published:** 2015-07-10

**Authors:** Borgný Katrínardóttir, José A. Alves, Hrefna Sigurjónsdóttir, Páll Hersteinsson, Tómas G. Gunnarsson

**Affiliations:** 1 Ecology Department, Icelandic Institute of Natural History, Gardabaer, Iceland; 2 South Iceland Research Centre, University of Iceland, Selfoss/Gunnarsholt, Iceland; 3 CESAM, University of Aveiro, Aveiro, Portugal; 4 Department of Life- and Environmental Sciences, University of Iceland, Reykjavik, Iceland; Australian National University, AUSTRALIA

## Abstract

Distinct preference of species for habitats is most often driven by long term differences in demographic rates between habitats. Estimating variation in those rates is key for developing successful conservation strategies. Stochastic events can interact with underlying variation in habitat quality in regulating demography but the opportunities to explore such interactions are rare. Whimbrels in Iceland show a strong preference for sparsely vegetated riverplains. Such habitats in Iceland face various threats, e.g., climate change, river regulation and spread of alien plant species. In this study we compared demographic parameters of breeding Whimbrels between riverplains and other habitats before, during and after volcanic eruption events to estimate the importance of the habitats for the species and the effect of ash deposit on breeding success. We found that an estimated minimum of 23% of the Icelandic population of Whimbrels and c. 10% of the world population of the species breed in riverplain habitats in Iceland. Whimbrels bred consistently at much higher densities in riverplain habitats than in other habitats and riverplains also had higher densities of pairs with fledglings although the proportion of successful breeders was similar between habitats. Predation by livestock may have had a considerable negative effect on breeding success on our study sites. Breeding was negatively affected by the volcanic activity, probably through the effects of ash on the invertebrate food supply, with breeding success being gradually worse closer to the eruption. Breeding success was equally affected by volcanism across habitats which differed in underlying habitat quality. This study gives an example of how populations can be regulated by factors which operate at different spatial scales, such as local variation in habitat quality and stochastic events which impact larger areas.

## Introduction

Habitat specialisation is one of the major factors contributing to species vulnerability to habitat loss [1]. Species which show preference for specific habitats are by definition disproportionately distributed between habitats, therefore factors affecting the preferred habitats can greatly affect specialist species. The variation in demographic parameters between preferred and other habitats will determine the effects of habitat loss on populations as habitat preference might not neccessarily translate into variation in demographic rates. Such effects can operate through density dependence, with reduced average fecundity through increased competition in preferred habitats (e.g. [2,3]). Estimating the variation in demographic rates between habitats that differ in apparent quality (assessed by preference of individuals towards different habitats) is therefore a key factor in estimating the effects of habitat loss on population demography. The variation in habitat quality is largely driven by factors such as hydrology, soil type and vegetation characteristics which relate to demography, e.g. through resource abundance, shelter, inter- and intraspecific interactions (e.g. [4]). Such factors can operate at different spatial as well as time scales and determine the duration of the suitability of particular patches for individual species, such as through vegetation succession and geomorphological processes. Stochastic factors, such as extreme weather events and natural hazards [5] can also affect habitat quality and demography (e.g. [6]) although the duration of those effects can vary greatly. Stable long-term habitat suitability and short-term stochastic events which affect habitat quality have the potential to interact in their effects on demography at different scales.

Many of the world‘s wader species (Charadrii) are currently in decline due to loss and degradation of habitats, often due to intensification of agriculture and wetland drainage [5]. It has been estimated that 48% of wader populations with known trends are declining [7], with especially severe declines being reported for populations using the East Asian-Australasian Flyway [8]. Iceland hosts internationally important numbers of several species, e.g.: Golden Plover (*Pluvialis apricaria*) (52%), Purple Sandpiper (*Calidris maritima*) (46%), Whimbrel (*Numenius phaeopus*) (40%) and Ringed Plover (*Charadrius hiaticula*) (32%) [9]. Approximately 250,000 Whimbrel pairs breed in Iceland, representing the bulk of the subspecies *N*.*p*. *islandicus* which also has much smaller populations in Greenland (50–100 pairs), the Faeroes (2,500 pairs) and the UK (500 pairs) [10]. The status of the large Icelandic population is believed to be stable [11] but the population in Shetland has suffered a major decline [12] and a decline has been reported in the Faeroes as well [11]. Although Icelandic Whimbrels are found in several habitats, e.g. heathland, wetland and grassland [13], they show strong habitat preference for sparsely vegetated riverplains [9]. The results of a three year comparison of one riverplain area and one heathland area in 1997–2000 showed that breeding success was much greater on the riverplain, possibly due to higher food supply and lower predation [14]. But studies encompassing both larger spatial and temporal scales, as well as short term effects of events influencing habitat quality are required to investigate the mechanistic links between habitat and fitness.

Riverplain habitats comprise about 8% of Iceland’s lowlands. [9]. They are most extensive along large glacial rivers, with regular floods in these areas keeping the vegetation structure at an early successional stage [15] and suitable for open-habitat species like the Whimbrel. In Iceland, five of the ten major catchments have been disrupted with dams and four others are being evaluated for potential water harnessing [16]. Damming of rivers for hydroelectric powerplants may disrupt seasonal flood regime and can alter the plant and animal communities in riverplains [15,17]. River regulation, along with disappearance of glaciers due to climate change [18] and spread of alien plant species (mainly *Lupinus nootkatensis*) [19], could cause dramatic changes in these areas in coming decades, resulting in taller and denser vegetation. These changes are likely to affect breeding Whimbrels in these areas as tall vegetation seems to negatively affect breeding success in the closely related species Long-billed Curlew (*Numenius americanus*) [20,21] and Whimbrels in Canada avoid habitats with encroaching woody vegetation [22].

Much of lowland Iceland is affected by volcanic activity and volcanic eruptions occur every 3–4 years [23]. Volcanos regularly emit large quantities of dust which is further redistributed by wind (e.g. [24,25]). In the long term, this dust seems to positively affect ecosystem productivity at large-spatial scales and is detected in the distribution and abundance of common birds across the country which occur in higher densities in areas with higher dust deposition rates [26]. But the immediate effects of eruptions on birds are poorly understood as opportunities to explore them are rare (but see [27–29]) and no studies have directly assessed the effect of eruptions on wader breeding parameters.

Another factor that greatly affects ecosystems in Iceland is grazing, but much of Iceland has been heavily grazed for centuries and grazing has likely played a role in extensive vegetational degradation and soil erosion since the country was settled more than 1100 years ago [30]. However, in the lowlands, grazing might also have had a part in creating and sustaining suitable open habitats for the large populations of waders in Iceland [31].

In this study we compared variation in breeding density, breeding success (measured as proportion of birds with chicks) and return rates of Whimbrels between riverplain habitats and grassland/heathland habitats in order to explore possible drivers for this preference and to estimate the relative importance of the threatened riverplain habitats for the species. If habitat preferences are based on higher fitness in the preferred habitat, then we predict that breeding success of Whimbrels in riverplains will be higher than in other habitats.

During the study, 2009–2011, two nearby volcanoes erupted and emitted large quantities of volcanic ash over the study areas, providing a unique opportunity to explore the immediate effects of ash deposition by testing the effect of distance from the eruption on Whimbrel breeding performance and to explore how volcanism affected different habitats.

## Methods

### Site access and study ethics

Access to study sites was granted by land owners of individual sites. Catching, ringing and handling of the Whimbrels was non-invasive, followed standard procedures and was permitted by the Icelandic Institute of Natural History (http://www.ni.is/). No additional animal care approval was required as all handling and sampling procedures performed in this study were covered by the ringing permit from the Icelandic Institute of Natural History and therefore not subject to special reviewing. The study did not involve any endangered species.

### Survey structure and study sites

The study took place in Southern Iceland in the summers of 2009–2011 in the largest lowland area in the country. In total, eight sites were surveyed, four riverplain sites and four sites in heathland/grassland areas, to attain measures of breeding density and breeding success (Table 1). Furthermore, on two of these sites (one riverplain and one grassland) the fate of nests and chicks of individually marked adults was studied in more detail (henceforward referred to as main sites and the other remaining six sites referred to as survey sites). Structural differences between habitat types have been described before [9]. The main riverplain site (63°43,150'N, 20°0,274'W) covered 0,9 km^2^ and characteristic plant species included mosses (bryophytes), crowberry (*Empetrum nigrum*), arctic rush (*Juncus arcticus*), and willows (*Salix* spp.). As is the case with much of lowland Iceland, the area was grazed by sheep and horses. The comparative main site (63°48,346'N, 20°9,576'W) was a mosaic of grassland and heathland, partially eroded and covering in total 1.4 km^2^. Common plants included bellardi bog sedge (*Kobresia myosuroides*), viviparous sheep's-fescue (*Festuca vivipara*), arctic fescue (*Festuca richardsonii*) and field horsetail (*Equisetum arvense*). This grassland site was also grazed by sheep and horses.

**Table 1:** Study sites.

Habitat	Type	Name	Coordinates	Area (km^2^)	Distance from eruption (km)
Riverplain	Main site	Smaratun	63°43,150'N, 20°0,274'W	0.9	22
	Survey site	Frodholtshjaleiga	63°44.987'N, 20°25.975'W	0.5	42
	Survey site	Saudholt	63°50.828'N, 20°39.998'W	0.45	58
	Survey site	Arnarbaeli	63°56.629'N, 21°12.659'W	0.6	86
Grassland/heathland	Main site	Hof	63°48,346'N, 20°9,576'W	1.4	33
	Survey site	Hvolsfjall	63°45.671'N, 20°11.915'W	0.6	33
	Survey site	Hadegisholt	63°55.924'N, 20°30.699'W	2.5	56
	Survey site	Minniborgir	64°04.924'N, 20°43.933'W	1.2	76

Names, coordinates and area of study sites. Also shown are the distances between study sites and Eyjafjallajokull

### Breeding density and breeding success across habitats

Density of breeding pairs was estimated by walking through study sites and mapping birds that exhibited behaviour indicating they were nesting in the area (e.g. were clearly alert, agitated and/or vocal). On the main sites, this estimate was also based on known nests and individually marked birds [32]. If there were two birds together that were not seen being aggresive towards each other, they were assumed to be a pair. Single territorial birds were also assigned the status of a pair and assumed that the mate was absent or incubating. In 2010 and 2011, counts were performed every two weeks, beginning in early June when peak arrival time has finished [33] until the end of the breeding season in late July. In 2009, counts for all sites were only attained for the fledging period (i.e. post-hatching).

Counts were usually conducted on the same time of day, in the afternoon and early evenings, due to diurnal differences in detectability [34]. The estimated proportion of birds with chicks was obtained by comparing the number of birds that were still present in late July, when the vast majority of nests should have hatched and the oldest chicks are about to fledge, with the number of birds in the same areas during the nesting period. This method has been used successfully for at least two related species to obtain estimates of large-scale breeding success [35,36].

### Demographic parameters on main sites

Nests were located on the main sites and eggs put in water to establish incubation stage (which can be estimated from the angle and flotation of the egg) [37]. Birds were caught on the nest using a tilting cage (RB60, http://www.moudry.cz/), individually marked with colour rings and released after standard measurements and feather samples for sexing had been taken [32].

Nests were visited twice a week to determine nest success or failure. Successful hatching was confirmed by finding chicks in or close to the nest, with remains indicative of hatching in the nest lining (small parts of eggshell or shell membrane) or by agitated adults nearby.

In 2010, motion-triggered cameras (Scoutguard SG560V in camouflage, HCO) were placed by nests at the main sites to monitor predation. The cameras were attached to poles and positioned facing north about two meters from the nests and as low as possible (5–10 cm from the ground) to avoid detection from predators. The cameras were programmed to take three pictures when triggered with a seven second interval and sensor level was set to high.

Chicks of marked pairs on the main sites were counted within a week of fledging to estimate fledging success. Marked pairs were monitored and watched from a distance, usually on more than one occasion, to estimate minimum brood-size. Mature chicks of unmarked birds on the main study sites were also included in calculations of final brood sizes for those adults. The movement of marked birds suggested that they stayed largely within their territories so it is unlikely that the inclusion of broods of unmarked birds might bias the data.

The return rate of marked adults between years was also compared between the main sites. As most adult waders are highly faithful to breeding sites (e.g. [38]), return rates reflect minimum surivial. Return rate can also be influenced by dispersal which can differ in relation to habitat-specific breeding success [39].

### Effects of volcanic eruptions

On the 14th of April 2010, Eyjafjallajökull (63°38,0'N 19°37,0'W) erupted after having been dormant since 1821 [23]. The eruption lasted for 39 days, emitting vast amounts of ash to the atmosphere and to nearby land following a gradient of ash deposition with distance from the volcano [25]. The eruption was located 22 and 33 km from the main riverplain and grassland sites respectively and survey sites were located between 33 and 86 km away. All study sites were west of the volcano and due to predominantly northerly winds during the eruption, they were not covered with as thick layer of ash as areas south and southeast of the volcano. This eruption provided a unique opportunity to explore the effects of moderate ash deposition on breeding success by comparing success between sites on a distance gradient from the volcano. On the 21st of May in 2011, a volcanic eruption started in Grímsvötn (64°25,12'N 17°19,48'W) lasting for a week, which added a smaller amount of ash to the study areas but its epicentre was located much further away from the study sites or between 152 and 196 km.

### Statistical analysis

Daily survival probability (DSR) of nests was calculated according to the Mayfield method [40,41] that corrects for exposure time of nests so not to overestimate hatching success due to nests that are found late in incubation compared to those found early on. Standard error of DSR was calculated according to Johnson [42] and comparisons of DSR were done according to Hensler and Nichols [43].

To investigate the differences in breeding density and density during chick rearing (i.e. breeding success) between habitats and years, we ran two separate generalized linear models with poisson error distribution, each having habitat, year and their interaction as explanatory variables. Linear regression models were used to explore large-scale (among sites) relationship between breeding density and breeding success. Chi-square tests were used to test between differences in proportions of pairs that hatched chicks between habitats, as well as for return rates of marked birds and number of fledged chicks on the main sites.

The effects of the volcanic eruption on breeding success on all the sites was tested with a generalized linear model with binomial error distribution, using the proportion of successful breeders (pairs with chicks) as the response variable and distance from the volcano, year (2010 and 2011) and habitat as explanatory variables.

All calculations and tests were done in the statistical software R (version 2.13.1, R Development Core Team).

## Results

### Breeding density and breeding success

**Fig 1:**
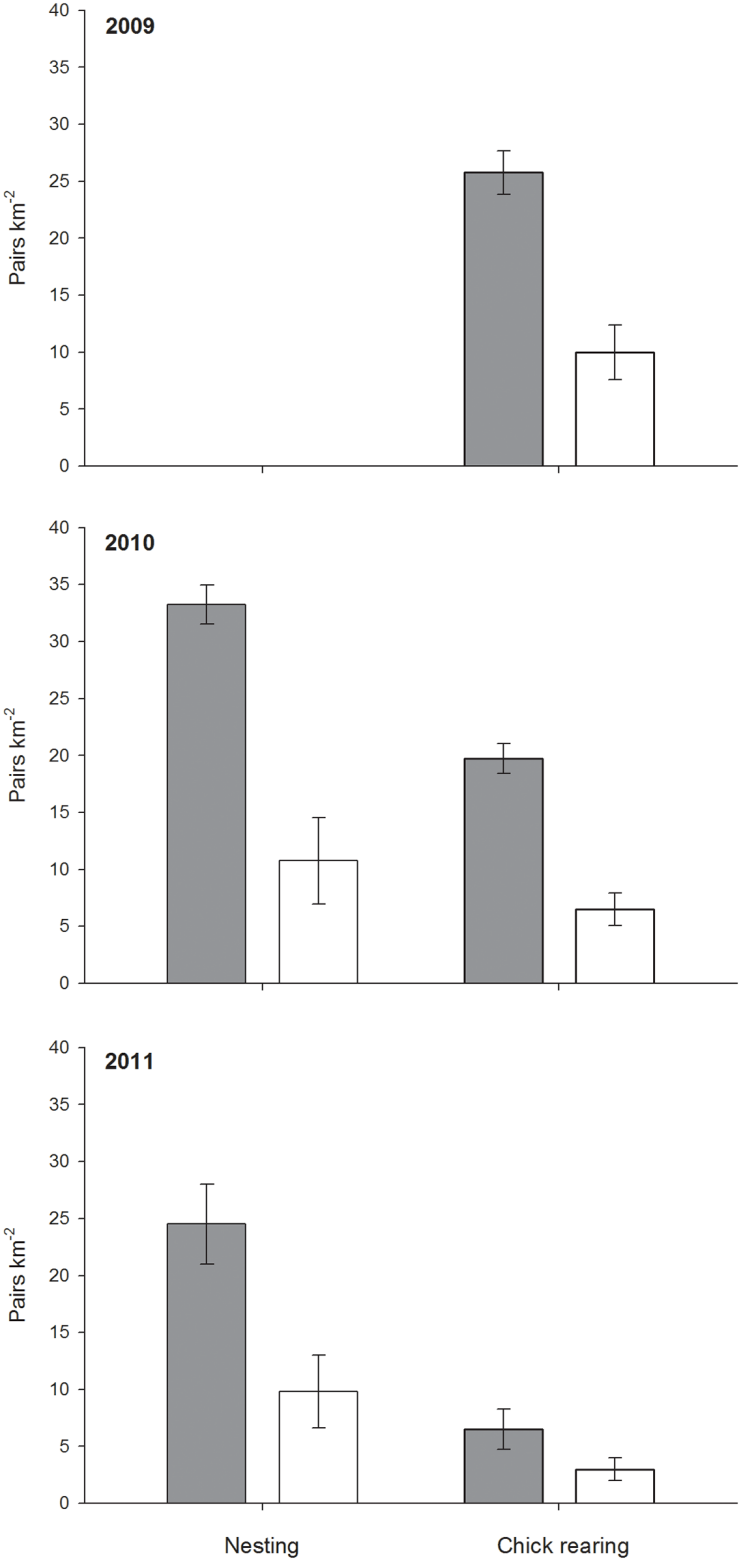
Density of breeding Whimbrels. Average (± SE) density of breeding Whimbrels between riverplain (grey) and grassland/heathland (white) sites during nesting 2010 and 2011, and during chick rearing between 2009 and 2011.

Mean density of nesting pairs on all the riverplain sites was 33.3±1.7 (SE) pairs/km^2^ in 2010 and 24.5±3.5 pairs/km^2^ in 2011 whereas on grassland/heathland sites it was 10.8±3.8 pairs/km^2^ in 2010 and 9.8±3.2 pairs/km^2^ in 2011 (Fig 1). Density of breeding pairs was significantly higher on the riverplain sites in both 2010 and 2011 and this difference was consistent across years (Fig 1, Table 2), despite densities being overall higher in 2010 than in 2011.

**Table 2:** Effects of habitat and year on density.

	Df	χ^2^	p
***Breeding density***			
Habitat	1	73.89	**<0.0001**
Year	1	4.87	**0.0273**
Habitat*Year	1	0.65	0.4213
***Density during chick rearing***			
Habitat	1	61.32	**<0.0001**
Year	2	66.34	**<0.0001**
Habitat*Year	2	0.72	0.6976

Results of generalized linear models, with density (breeding density and density during chick rearing) as the response variables, and habitat and year as explanatory variables.

Density of pairs with chicks on riverplain sites was 25.8±1.9 pairs/km^2^ in 2009, 19.8±1.3 pairs/km^2^ in 2010 and 6.5±1.8 pairs/km^2^ in 2011 whereas in grassland/heathland sites it was 10.0±2.4 pairs/km^2^ in 2009, 6.5±1.4 pairs/km^2^ in 2010 and 3.0±1.0 pairs/km^2^ in 2011 (Fig 1). In all three years the average density of pairs with chicks was consistently higher in riverplain habitats (Fig 1, Table 2), with overall numbers being significantly lower across years.

There was no large scale relationship between breeding density and breeding success in 2010 (linear regression: y = 0.864–0.008x; R^2^ = 0.155; p = 0.181) nor in 2011 (linear regression: y = 0.531–0.011x; R^2^ = 0.168; p = 0.172). On average, the proportion of successful pairs in 2010 was 59% on riverplain sites and 67% on grassland/heathland sites (χ^2^ = 0.087; df = 1; p = 0.768). In 2011, this proportion was 28% on the riverplain sites and 40% on grassland/heathland sites(χ^2^ = 0.128; df = 1; p = 0.721).

### Relationship between volcanism and whimbrel demography

**Fig 2:**
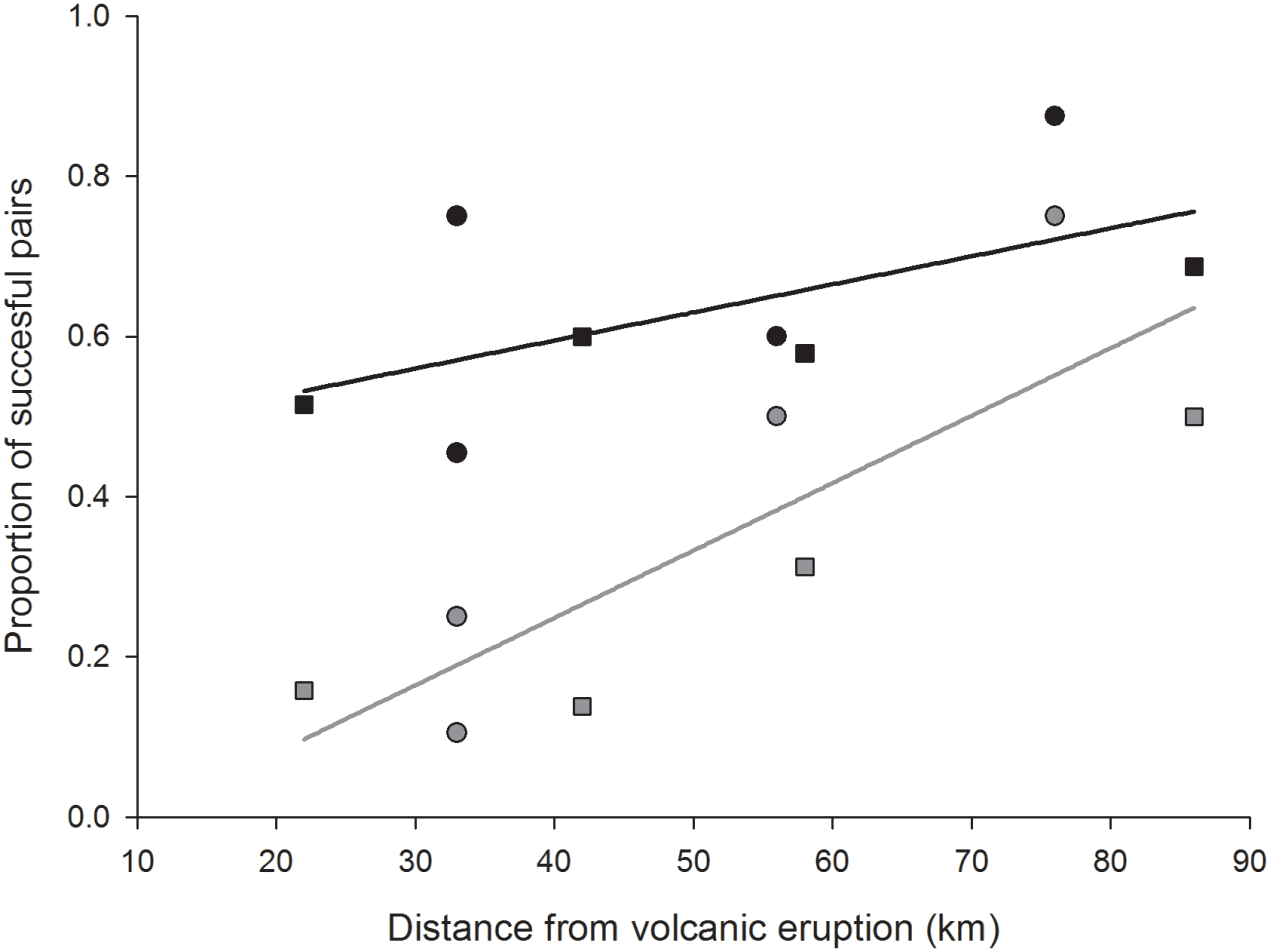
Effect of distance to Eyjafjallajokull volcano on the proportion of successful pairs. 2010 is shown with black symbols & line (y = 0.0035x + 0.455) and 2011 with grey symbols & line (y = 0.0084x−0.088). Riverplains are shown with squares and grassland/heathland with circles.

Breeding densities and the densities of pairs with chicks were considerably reduced after 2009 and 2010 (Fig 1). There was significant effect of both distance from volcano and year on the proportion of successful breeders but not of habitat which was therefore omitted from the final model (Table 3). There was also a significant effect of the interaction between distance and year between 2010 and 2011 with the drop in success more pronounced nearer the volcano (Fig 2, Table 3).

**Table 3:** Effects of distance from volcano and year on breeding success.

	Estimate	Std. Error	z value	P
Intercept	5398.334	1421.119	3.799	0.0002
Distance	-49.771	25.007	-1.990	0.0466
Year	-2.686	0.707	-3.799	0.0002
Distance*Year	0.025	0.012	1.991	0.0465

Results of generalized linear model, with the proportion of successful breeders (pairs with chicks) as the response variable and distance from the volcano and year (2010 and 2011) as explanatory variables.

### Hatching and fledging success on main sites

Estimated hatching success and daily survival probability (DSR) did not differ between the main riverplain and grassland sites in 2009 nor 2010 and neither was there a significant difference between years within each habitat (Table 4). Too few nests were found in 2011 to allow comparison with previous years.

**Table 4:** Nest success and daily survival of nests.

	Riverplain			Grassland			
Year	Hatched nests (%)^a^	Hatched nests (%)^b^	Daily survival (SE)	Hatched nests (%)^a^	Hatched nests (%)^b^	Daily survival (SE)	
**2009**	33 (n = 12)	19	0.945 (0.019)	36 (n = 14)	17	0.941 (0.019)	Z = 0.179; p = 0.858
**2010**	47 (n = 15)	29	0.958 (0.015)	33 (n = 18)	15	0.936 (0.018)	Z = 0.938; p = 0.348
			Z = 0.516; p = 0.606			Z = 0.175; p = 0.861	

Observed and estimated nest success and daily survival of nests on main study sites.

^a^Observed nest success (successful nests/all nests).

^b^Hatched nests according to the Mayfield method, nesting period of 29 days used in calculations.

The most common cause of nest and egg losses was predation with 126 eggs of 197 being depredated on both main sites in 2009 and 2010. Six eggs were abandoned and 2 eggs were infertile. In 2010, cameras monitored a total of 15 nests (8 on the riverplain site and 7 on the grassland site) for a varied length of time (range 1–20 days, mean = 8.6 days). The nest cameras recorded a total of 13 predation events. On the riverplain site they were solely produced by livestock, sheep ate eggs from nests on four instances and on two occasions horses were responsible (although they didn’t seem to eat much of the eggs). On the grassland site, Arctic skuas (*Stercorarius parasiticus*, 3), Arctic fox (*Alopex lagopus*, 1) and sheep (3) depredated the nests.

Average brood size just before fledging on the main riverplain site in 2010 was 1.3 chicks/pair (n = 7) and 1.5 chicks/pair (n = 6) on the grassland site which was not significantly different (Wilcoxon rank sum test. W = 16.5, p = 0.5).

On riverplain site, 11 pairs produced a total of 19 chicks of which 21% (4) fledged, while on the grassland site, 14 pairs produced 22 chicks and 32% (7) of them were assumed to have fledged (Table 5) with no significant difference in proportion of chicks that fledged between the main study areas (χ^2^ = 0.178; df = 1; p = 0.67). No chicks were confirmed to have fledged in 2011 on either site.

**Table 5:** Fledging success.

	No. of pairs	No. of chicks	No. of fledged chicks	Chick survival	Fledged chicks/pair
Riverplain	11	19	4	0.21	0.36
Grassland	14	22	7	0.32	0.5

Fledging success on main study sites in 2010.

### Adult return rate

On the riverplain site, 70% of the birds ringed in 2009 returned in 2010, whereas 78% returned to the grassland site. Of ringed birds present in 2010, 63% were resighted on the riverplain site in 2011 while 57% returned on the grassland site. Overall, there was no difference in return rates between sites nor between years (Table 6).

**Table 6:** Return rates.

Year	Riverplain		Grassland
**2010**	70 (7/10)	χ2 = 0.148; df = 1; p = 0.701	78 (7/9)
	χ2 = 0.136; df = 1; p = 0.713		χ2 = 1.155; df = 1; p = 0.283
**2011**	63 (12/19)	χ2 = 0.150; df = 1; p = 0.698	57 (12/21)

Return rates of Whimbrels to the main study sites in %. Proportions of ringed birds the previous year shown in parentheses.

## Discussion

### Demography in different breeding habitats

Breeding densities of Whimbrels in riverplain areas in the southern part of Iceland are among the highest recorded worldwide. In this study, the average breeding density on the riverplain sites was 29 pairs/km^2^ whereas a previous study found a stable breeding density on a riverplain site over a period of three years around 40–45 pairs/km^2^ [14]. The highest density in a heathland area in Shetland was 21.4 pairs/km^2^ [44] and in Churchill, Manitoba, 11.5 pairs/km^2^ were recorded in a hummock-bog habitat for the *hudsonicus* subspecies [45]. Despite this high breeding density, we found no apparent relationship between breeding density and hatching success which was similar for riverplain areas and grassland/heathland habitats. This is contrary to an earlier study where an estimated 61–100% of nests hatched each year on the riverplain but only 1–19% on a heathland site [14]. In Canada, hatching success was also highest in the most densely populated habitat [45]. Hatching success among waders is known to vary greatly among species, in time and between areas [46] and documented hatching success for Whimbrels in other countries ranges from 14–86% [22,44,45,47–49].

Chick survival from hatching to fledging was also rather poor when compared to an earlier study, which found average survival from hatching to fledging to be 52% on the riverplain site while it was 38% on the heathland site [14].

In this study 13 egg predation events were recorded and in most cases sheep were responsible. Egg and chick eating by sheep is a known phenomenon [50,51] that might stem from mineral deficiency [51]. Mammalian predators are common predators of wader nests [52] and Arctic foxes were suspected to have depredated several nests in the current study and an earlier study [14]. However, egg predation by livestock could be an important factor driving local predation effects and may have had considerable impact at the main study sites. Futhermore, no natural predator was recorded removing eggs in the riverplain site, therefore, in the absence of livestock, hatching success might have been higher on the riverplain in this study, as previously recorded [14]. Grazing can have various effects on ground nesting birds. These effects vary in relation to timing, density and type of grazing animals and can be both negative and beneficial [53,54]. In Iceland, most lowland habitats where waders generally breed at high densities are maintained open by grazing so there is likely some balance between the positive effects grazing has on habitat suitability, and predation rates and other negative effects. Moderate grazing could prove to be an important factor in keeping the vegetation height in riverplain areas suitable for Whimbrels in the absence of floods due to river impoundments.

There was no difference in minimum survival rate (return rate) of marked adults between the main study sites. The return rates reported here are similar to the results from Gunnarsson‘s study [14] where annual return rates were 60–82%. In Canada, Whimbrels seemed to show more site tenacity towards the habitat with higher hatching success [45]. The return rates recorded in Iceland are somewhat lower than those found for Whimbrels in Shetland [44] where return rates were 87% for males and 68% for females. In 2011, several birds were only seen once and few pairs seemed to attempt breeding on the main study sites that year. The spring and early summer of 2011 was cold and another eruption started in Grimsvotn, S-Iceland, adding ash to the amount already present from the previous year's Eyjafjallajokull eruption.

One of the main drivers of difference in breeding output of birds is food abundance [55] and in an earlier study, the invertebrate supply in a riverplain area was much higher than in a heathland habitat [14]. In this study however, volcanic eruptions probably affected the invertebrate abundance and may have done so disproportionally between the main study sites as the riverplain site was closer to the more relevant eruption in 2010.

The Icelandic Institute of Natural History has monitored trends in Lepidoptera and Trichoptera spp. with light traps in the vicinity of Eyjafjallajokull since 1995. One trap site is located less than 4 km from the main riverplain site. The post-eruption data is still being processed but it is clear that there was a complete collapse for many species in 2010 as well as a delayed peak of abundance in some cases (Olafsson, E. Pers. comm.). It is therefore likely that invertebrate prey of Whimbrels was affected by the volcanic eruptions, which in turn could have negatively affected chick survival.

Volcanic ash is known to have adverse effect on many invertebrate groups although the effect varies between taxons and life stages [56–60]. Counts of the forest birds on the Lesser Antillean island of Montserrat indicated a decrease in numbers following major ashfall but the effect seemed to be short-lived and populations recovered quickly in subsequent years [28].

### Drivers of variation in breeding density

Whimbrels showed consistently higher breeding density on riverplains, at all stages of the breeding cycle. However, the results presented here and by Grant [44] indicate no clear relationship between breeding density and breeding success.

Higher nest density might lead to higher predation pressure, (e.g. [61–63]) as seems to be the case with density dependent predation on Snowy plover (*Charadrius alexandrinus*) nests in California [64]. It is also possible that high density in riverplains has lead to such severe competition that differences in breeding success between habitats has disappeared through density dependence (e.g. [3]).

But high density could also be beneficial when it comes to defending against aerial predators. Whimbrels are very apt flyers and aggressively mob much larger birds [65] and higher density means more birds joining chases [14]. In an experimental study with artificial nests, predation was significantly lower where the aggressive Lapwings (*Vanellus vanellus*) and Curlews (*Numenius arquata*) were present and defended against aerial predators [62].

High breeding density in riverplains might also be a response to resource abundance being higher there than in other habitats (e.g. [66–71]) although it is uncertain whether birds adjust their territory size to available resources or if they are unable to fend off competitors for the preferred habitats.

For many birds, like most waders, which show strong adult philopatry [46] and natal philopatry to a certain degree [39,72–74] flexibility in site selection and spatial knowledge of site quality are probably limited, especially for waders that breed in subarctic and arctic areas and have therefore very limited time to complete their breeding cycle [75].

Due to these limitations it seems probable that higher density in riverplains is achieved through breeding success and/or survival being higher there on average. Whimbrels are long lived birds and the evolution of habitat selection probably only requires slight differences in productivity or survival over long time periods. It can therefore not be excluded that Whimbrels on riverplains have a higher individual fitness on average that went undetected in this study.

### Consequences of habitat selection and volcanic eruptions for Whimbrel demography

Almost half of the estimated world population of Whimbrels breeds in Iceland and evidence suggests that riverplains are under threat so estimating the relative importance of this key habitat for population demographics is important for successful conservation. Riverplains in Iceland constitute around 8% of Iceland‘s lowlands [9]. In this study, the breeding density on riverplains was on average 29 pairs/km^2^. The Icelandic population is thought to consist of 250 thousand pairs [10] and from that we can roughly estimate that 23% of the Icelandic population breeds in riverplain areas, which means that c. 10% of the world population breeds in this habitat in Iceland. However, more long-term studies of demography and larger scale comparison of variation in resource abundance between habitats are needed to better identify the processes underpinning the preference of Whimbrels for riverplains.

The two volcanic eruptions which occurred half-way through the study provided an opportunity to assess the short-term effect of volcanism on Whimbrel demography. A clear positive relationship between distance to volcano and breeding success, suggests a link with the amount of ash deposition. A likely driver of this relationship is the negative effect which volcanic ash has on the invertebrate food stock of birds although it seems that these effects are generally short-lived [28,60] and there is a positive relationship between long-term ash deposition rates and wader abundance in Iceland [26]. The magnitude of ash plays a role. The high ash volume during and close to eruptions appears to have an immediate and significant impact, but in the long-term, redistribution of volcanic material by wind can have fertilizing effects on ecosystems [76,77]. There is no indication that these volcanic eruptions affected the different Whimbrel habitats disproportionally but rather operated independently of habitat. This study gives an example of a system where demography is both affected by habitat type and stochastic events which operate independently of habitat types. This is likely to be a very common scenario in animal populations but is rarely reported. Long-term difference in habitats is a key driver of variation in breeding density and, presumably, fitness, which plays a role in selection driving habitat preferences. The effect of stochastic events on habitat selection processes will thus depend on the frequency of extreme events and how they interact with habitat types.

## Supporting Information

S1 File Density of Whimbrels. Density during nesting and chick rearing for Whimbrels on each of the study sites.
(PDF)

S2 File Breeding success in relation to distance from volcano. Successful and unsuccessful pairs on each of the study sites and the distance between sites and Eyjafjallajokull
(PDF)

S3 File Nest data. Information about nests found on the main study sites.
(PDF)
