# Supplementary material for: The Effects of Habitat Type and Volcanic Eruptions on the Breeding Demography of Icelandic Whimbrels Numenius phaeopus
Source: PLoS One. 2015 Jul 10;10(7):e0131395. doi: 10.1371/journal.pone.0131395 (PMC4498681; doi:10.1371/journal.pone.0131395)
Supplement: S1 File — Density during nesting and chick rearing for Whimbrels on each of the study sites. (PDF) [file pone.0131395.s001.pdf]

Site	Habitat	Year	Density during nesting (Pairs/km2)	Density during chick rearing (Pairs/km2)
Smaratun	Riverplain	2009	N/A	25
Frodholtshjaleiga	Riverplain	2009	N/A	31
Saudholt	Riverplain	2009	N/A	22
Arnarbaeli	Riverplain	2009	N/A	25
Minna-Hof	Grass/heathland	2009	N/A	17
Hvolsfjall	Grass/heathland	2009	N/A	8
Hadegisholt	Grass/heathland	2009	N/A	7
Minniborgir	Grass/heathland	2009	N/A	8
Smaratun	Riverplain	2010	33	17
Frodholtshjaleiga	Riverplain	2010	30	18
Saudholt	Riverplain	2010	38	22
Arnarbaeli	Riverplain	2010	32	22
Minna-Hof	Grass/heathland	2010	22	10
Hvolsfjall	Grass/heathland	2010	8	6
Hadegisholt	Grass/heathland	2010	5	3
Minniborgir	Grass/heathland	2010	8	7
Smaratun	Riverplain	2011	19	3
Frodholtshjaleiga	Riverplain	2011	29	4
Saudholt	Riverplain	2011	32	10
Arnarbaeli	Riverplain	2011	18	9
Minna-Hof	Grass/heathland	2011	19	2
Hvolsfjall	Grass/heathland	2011	8	2
Hadegisholt	Grass/heathland	2011	4	2
Minniborgir	Grass/heathland	2011	8	6
