# Supplementary material for: The Effects of Habitat Type and Volcanic Eruptions on the Breeding Demography of Icelandic Whimbrels Numenius phaeopus
Source: PLoS One. 2015 Jul 10;10(7):e0131395. doi: 10.1371/journal.pone.0131395 (PMC4498681; doi:10.1371/journal.pone.0131395)
Supplement: S2 File — Successful and unsuccessful pairs on each of the study sites and the distance between sites and Eyjafjallajokull (PDF) [file pone.0131395.s002.pdf]

Site	Habitat	Year	Dist. from volcano (km)	Successful pairs/km2	Unsuccessful pairs/km2	Proportion of successful pairs
Smaratun	Riverplain	2010	22	17	16	0.515
Frodholtshjaleiga	Riverplain	2010	42	18	12	0.6
Saudholt	Riverplain	2010	58	22	16	0.579
Arnarbaeli	Riverplain	2010	86	22	10	0.688
Hof	Grass/heatland	2010	33	10	12	0.455
Hvolsfjall	Grass/heathland	2010	33	6	2	0.75
Hadegisholt	Grass/heathland	2010	56	3	2	0.6
Minniborgir	Grass/heathland	2010	76	7	1	0.875
Smaratun	Riverplain	2011	22	3	16	0.158
Frodholtshjaleiga	Riverplain	2011	42	4	25	0.138
Saudholt	Riverplain	2011	58	10	22	0.313
Arnarbaeli	Riverplain	2011	86	9	9	0.5
Hof	Grass/heathland	2011	33	2	17	0.105
Hvolsfjall	Grass/heathland	2011	33	2	6	0.25
Hadegisholt	Grass/heathland	2011	56	2	2	0.5
Minniborgir	Grass/heathland	2011	76	6	2	0.75
