# Supplementary material for: The Effects of Habitat Type and Volcanic Eruptions on the Breeding Demography of Icelandic Whimbrels Numenius phaeopus
Source: PLoS One. 2015 Jul 10;10(7):e0131395. doi: 10.1371/journal.pone.0131395 (PMC4498681; doi:10.1371/journal.pone.0131395)
Supplement: S3 File — Information about nests found on the main study sites. (PDF) [file pone.0131395.s003.pdf]

Year	Site	Habitat	No. eggs when found	Final no. of eggs	No. hatched	Predicted hatching	Hatching confirmed	Nest days	Fail reason
2009	Smaratun	Riverplain	4	4	0	21.6.2009		1	Predated
2009	Smaratun	Riverplain	2	N/A	0	22.6.2009		4.5	Predated
2009	Smaratun	Riverplain	2	4	0	25.6.2009		10	Predated
2009	Smaratun	Riverplain	2	5	0	21.6.2009		14.5	Predated
2009	Smaratun	Riverplain	3	2	2		20.6.2009	19	
2009	Smaratun	Riverplain	3	3	0	22.6.2009		11.5	Predated
2009	Smaratun	Riverplain	1	N/A	0	23.6.2009		4	Predated
2009	Smaratun	Riverplain	2	4	3		6.7.2009	27	
2009	Smaratun	Riverplain	4	4	4		26.6.2009	11	
2009	Smaratun	Riverplain	3	3	3		13.7.2009	23	
2009	Smaratun	Riverplain	4	3	0	14.7.2009		20	Predated
2009	Smaratun	Riverplain	3	N/A	0	13.7.2009		1	Predated
2009	Minna-Hof	Grassland	4	4	0	21.6.2009		4	Predated
2009	Minna-Hof	Grassland	4	4	4		21.6.2009	19	
2009	Minna-Hof	Grassland	4	4	0	16.6.2009		8.5	Predated
2009	Minna-Hof	Grassland	4	4	0	23.6.2009		8.5	Abandoned
2009	Minna-Hof	Grassland	4	4	4		20.6.2009	17	
2009	Minna-Hof	Grassland	4	4	0	15.6.2009		3.5	Predated
2009	Minna-Hof	Grassland	1	N/A	0	2.7.2009		3.5	Predated
2009	Minna-Hof	Grassland	4	2	2		11.7.2009	24	
2009	Minna-Hof	Grassland	4	1	1		9.7.2009	14	
2009	Minna-Hof	Grassland	2	N/A	0	26.6.2009		4	Predated
2009	Minna-Hof	Grassland	4	4	0	19.6.2009		4	Predated
2009	Minna-Hof	Grassland	4	3	3		27.6.2009	23	
2009	Minna-Hof	Grassland	3	3	0	27.6.2009		8.5	Predated
2009	Minna-Hof	Grassland	3	4	0	4.7.2009		10	Predated
2010	Smaratun	Riverplain	3	N/A	0			2.5	Predated
2010	Smaratun	Riverplain	4	4	0	15.6.2010		7.5	Predated
2010	Smaratun	Riverplain	4	4	4	18.6.2010	18.6.2010	24	
2010	Smaratun	Riverplain	4	4	0	18.6.2010		17.5	Predated

2010	Smaratun	Riverplain	3	7	0	22.6.2010		20	Predated
2010	Smaratun	Riverplain	4	4	4	18.6.2010	13.6.2010	16	
2010	Smaratun	Riverplain	4	4	4	15.6.2010	17.6.2010	15	
2010	Smaratun	Riverplain	3	3	0	26.6.2010		7	Predated
2010	Smaratun	Riverplain	4	1	1	11.7.2010	9.7.2010	25	
2010	Smaratun	Riverplain	2	2	0	8.7.2010		14.5	Predated
2010	Smaratun	Riverplain	4	3	3	22.6.2010	18.6.2010	3	
2010	Smaratun	Riverplain	4	3	3		26.6.2010	8	
2010	Smaratun	Riverplain	4	4	0	14.7.2010		12	Predated
2010	Smaratun	Riverplain	4	3	0	18.7.2010		4	Predated
2010	Smaratun	Riverplain	4	4	4		15.6.2010	13	
2010	Minna-Hof	Grassland	4	4	4		12.7.2010	26	
2010	Minna-Hof	Grassland	4	4	4		6.7.2010	13	
2010	Minna-Hof	Grassland	4	2	2	12.7.2010		8	
2010	Minna-Hof	Grassland	4	1	0	9.7.2010		23	Predated
2010	Minna-Hof	Grassland	3	N/A	0	21.6.2010		2	Predated
2010	Minna-Hof	Grassland	3	3	0	15.6.2010		4.5	Predated
2010	Minna-Hof	Grassland	1	N/A	0	28.6.2010		1	Predated
2010	Minna-Hof	Grassland	3	N/A	0	22.6.2010		2	Predated
2010	Minna-Hof	Grassland	3	3	0	23.6.2010		5	Predated
2010	Minna-Hof	Grassland	4	4	0	18.6.2010		5	Predated
2010	Minna-Hof	Grassland	4	4	4		22.6.2010	23	
2010	Minna-Hof	Grassland	3	2	0	6.7.2010		20	Predated
2010	Minna-Hof	Grassland	3	3	0	1.7.2010		11	Predated
2010	Minna-Hof	Grassland	4	4	4		18.7.2010	21	
2010	Minna-Hof	Grassland	2	2	0	24.7.2010		1	Abandoned
2010	Minna-Hof	Grassland	3	3	0	19.6.2010		17.5	Predated
2010	Minna-Hof	Grassland	4	3	0	21.7.2010		0.5	Predated
2010	Minna-Hof	Grassland	4	4	4		1.7.2010	4	
